# Systematic review and meta-analysis of the impact of diabetes mellitus on chronic venous insufficiency

**DOI:** 10.1590/1677-5449.202500062

**Published:** 2025-07-28

**Authors:** Alex Carlos Ferreira de Castro, Anderson Veiga Barbosa, David Fonseca Lima, João Vitor Rodrigues Vidal, José Maciel Caldas Reis, Saul Rassy Carneiro

**Affiliations:** 1 Universidade Federal do Pará UFPA, Belém, PA, Brasil.; 2 Universidade do Estado do Pará UEPA, Belém, PA, Brasil.

**Keywords:** vascular complications, diabetes mellitus type 2, occurrence

## Abstract

The systematic review with meta-analysis aimed to investigate the occurrence of Chronic Venous Insufficiency (CVI) in patients with Type II Diabetes Mellitus (T2DM). The guiding research question was structured using the PECOT framework (P: population; E: exposure; C: comparison; O: outcome; T: type of study), and the risk of bias was assessed using the ROBIS-I tool. A total of four studies were included in the analysis. The findings revealed that the prevalence of CVI in patients with T2DM was 55%, with a prevalence ratio of 1.51 (95% CI: 1.01 to 2.26). These results suggest that individuals with T2DM have a significantly higher prevalence of CVI compared to those without the condition. However, the review identified several limitations, such as the scarcity of longitudinal studies and variability in the diagnostic criteria for CVI among the included studies. The authors conclude that further research is warranted to address these gaps and deepen understanding of the relationship between T2DM and CVI.

## INTRODUCTION

Diabetes mellitus (DM) and chronic venous insufficiency (CVI) constitute public health problems of increasing relevance, given their high prevalence and the complex pathophysiologic interactions they share.^1-3^ DM is defined as a chronic dysfunction of glucose metabolism, caused by abnormalities of insulin activity or production, and is associated with many systemic complications, including cardiovascular diseases, nephropathies, and neuropathies, with negative impacts on individuals’ quality of life.^4^ The microvascular and macrovascular complications associated with DM are not exclusively caused by the prolonged hyperglycemia, but also by complex underlying mechanisms, such as inflammation, endothelial dysfunction, and oxidative stress, which contribute to vascular damage and disease progression.^1,4,5^

In turn, CVI is characterized by functional incapacity of veins to provide adequate return of blood from the lower limbs to the heart, resulting in symptoms such as edema, pain and, in more advanced cases, venous ulcers.^6,7^ The etiology of CVI is multifactorial, involving risk factors such as advanced age, obesity, family history and prolonged periods of immobility, whether sitting or standing.^7,8^ This condition affects millions of people worldwide, with prevalence that is increasing because of population aging and is consistent in many countries.^8-14^ It is estimated that the global prevalence of CVI ranges from 10 to 30% of the adult population, with higher frequency in women than men, at an approximate proportion of 2:1.^9-14^ Epidemiological data from Europe and the United States indicate that the prevalence of CVI in adults aged 30 to 70 years ranges from 5 to 15%, among whom around 1% of cases progress to ulcerative complications, affecting more than 7 million people and causing around 70 to 90% of lower limb ulcers.^15,16^

In Brazil, there is a dearth of studies of the prevalence and incidence of CVI. However, data from Brazil’s Unified Health System (SUS - Sistema Único de Saúde) health information systems for 2021 to 2024 contain 359,603,424 records showing related vascular conditions (ICD-10: I82.9, I80.3 and I74.4).^17^

Although both DM and CVI are conditions frequently encountered in clinical practice, there is a significant gap in the literature regarding their association with each other. Few studies have conducted comparative explorations of these two diseases and there is a dearth of data examining their similarities and combined effects on exacerbation of circulatory system dysfunctions. The objective of this systematic review and meta-analysis is to explore the association between DM and CVI, investigating the incidence of CVI in patients with DM and elucidating possible pathological interactions that contribute to development and progression of these conditions.

## METHODS

This systematic review with meta-analysis adheres to the recommendations of the Preferred Reporting Items for Systematic Reviews and Meta-Analyses (PRISMA) and is registered with the International Prospective Register of Systematic Reviews (PROSPERO) systematic reviews database under ID: CRD42024500961, to avoid unintentional publication duplication. The eligibility criteria are observational cross-sectional, case-control, and cohort studies, with no limits to publication date. The research question was formulated using the PECOT criteria (P: population; E: exposure; C: comparison; O: outcome; T: study type), as follows, population: adults over the age of 18; exposure: people with DM; comparison: people without DM; outcome: progression to chronic venous insufficiency; study type: observational cross-sectional, case-control, or cohort studies.

Studies with no comparison, literature and scoping reviews, and case series were excluded. There were no limitations regarding publication language. Studies were identified on the PubMed, EMBASE, and LILACS databases. Search strings comprised all of the keywords (''Diabetes mellitus'', ''complications'', ''Chronic venous insufficiency'', ''comorbidities'', and ''association'') combined with the Boolean operators "AND" and/or "OR" and were conducted independently by two reviewers and then their results were compared. Duplicate articles were excluded and then titles and abstracts were analyzed and those that met the inclusion criteria were selected for reading of the full texts. Any disagreements were discussed to establish consensus, with participation by a third evaluator.

Data were tabulated and analyzed using STATA 18.0 for random effects models and effect sizes were calculated from the effect on odds ratios. The proportion of CVI occurrence was calculated with a 95% confidence interval. Statistical heterogeneity between studies related to the effects of treatment was assessed using the inconsistency index (I^2^), TAU2 statistic, and Q index.

Risk of bias was assessed using the ROBINS-I tool, as recommended by the Cochrane collaboration (Supplementary Table 1, on-line only). The following domains were analyzed: bias due to confounding factors; bias in selection of participants into the study; bias in classification of interventions; bias due to deviations from intended interventions; bias due to missing data; bias arising from measurement of the outcome; bias in selection of the reported result; and overall bias. The quality of evidence was assessed independently by two reviewers using the Grading of Recommendations Assessment, Development and Evaluation (GRADE) system. Any disagreements were discussed until a consensus was achieved, with a third evaluator participating.^18^ The risks of bias of each of the studies analyzed are shown in Supplementary Table 1.

## RESULT

The online data search strategy identified 1,183 articles with application of the keywords ''Diabetes mellitus'', ''complications'', ''Chronic venous insufficiency'', ''comorbidities'', and ''association''. After extensive screening of titles and abstracts, 1,165 articles were excluded. A total of 18 articles were selected for full text reading and 10 of these were excluded because their central focus was not on the diseases of interest. Two of the remaining eight studies were then excluded because they only included patients with CVI and had not screened patients to differentiate between groups. Additionally, one article was excluded because there was little data on the number of patients with DM and the proportion with venous insufficiency, making it of little relevance to the review. Finally, one further article was excluded because the full text was not accessible, after a request addressed to the lead author had been unsuccessful.

**Figure 1:**
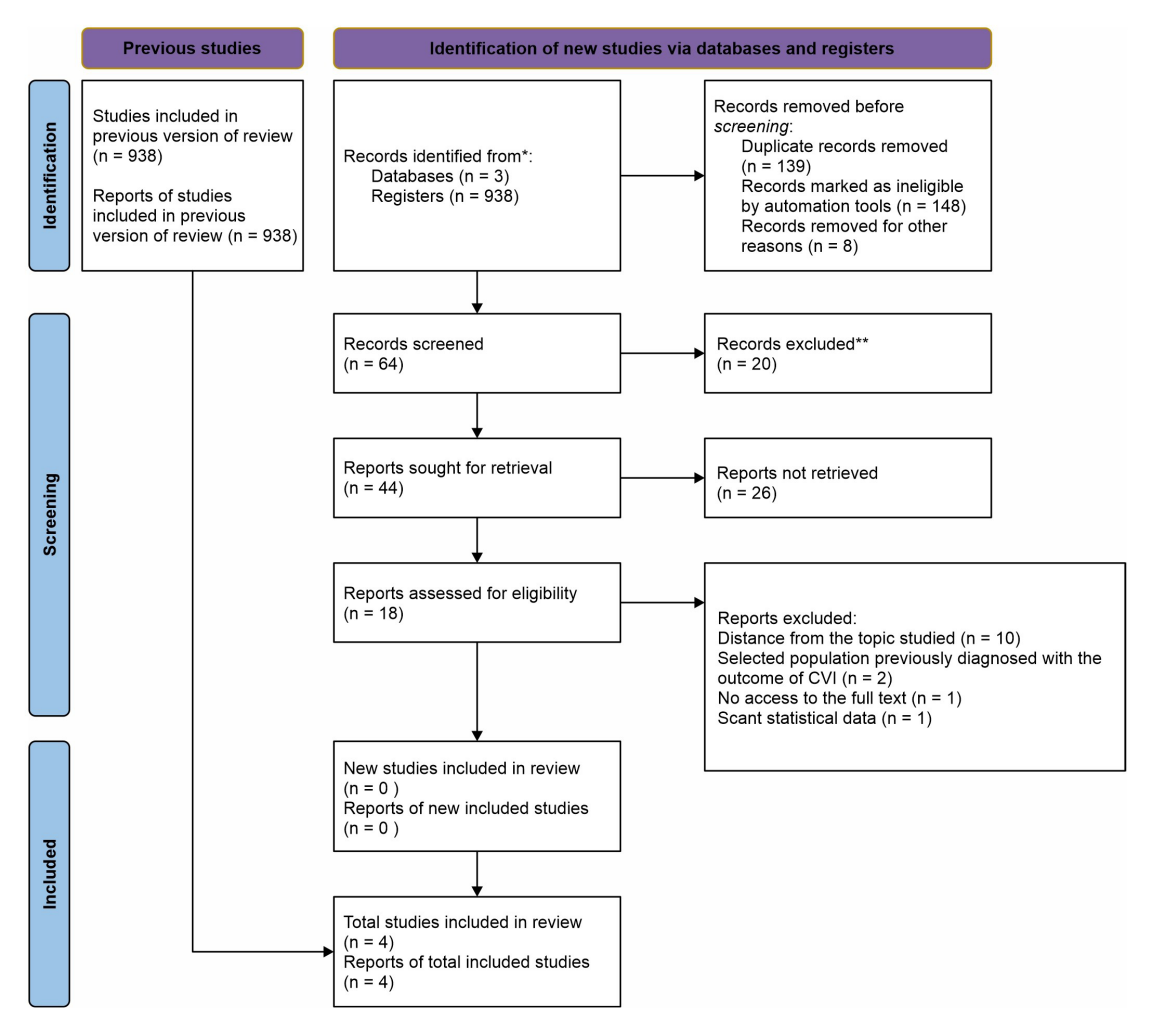
PRISMA (Preferred Reporting Items for Systematic Reviews and Meta-Analyses) study inclusion flow diagram, illustrating identification of studies, stages at which studies were excluded, and the reasons for exclusion. CVI = chronic venous insufficiency; *consider, if possible, reporting the number of records identified in each database or record searched (rather than the total number in all databases/records); **if automation tools were used, indicate how many records were deleted by a human and how many were deleted by automation tools.

As such, a total of four studies were included in this review; three cross-sectional studies and one prospective cohort study. However, all of them did compare the presence of CVI among patients with DM. The PRISMA diagram for this review is shown in Figure 1.

The period covered by the analyses reviewed ranged from 2009 to 2023 and they were conducted at centers in Serbia, Sweden, Iran, and Germany. The study by Matic et al.^3^ enrolled 174 patients who underwent analysis. Nine of these were excluded either because of lack of consent or because of missing data on patient records, leaving a total of 162 patients analyzed, 11 of whom had CVI and DM. A study by Forssgren and Nelzén^19^ reported similar results, with an initial sample of 621 participants. After exclusion of tests and dropouts from follow-up, 207 participants were analyzed, 68 of whom had DM and eight of whom had both DM and CVI concomitantly. The analyses reported by Babaei et al.^20^ demonstrated significant data, including a total of 1,176 participants. The statistical data showed that 165 of these people had DM, 71 of whom also had CVI. At the same time, the data reported by Meinel et al.^21^ also yielded relevant insights, with 71 patients with DM, 21 of whom had CVI and DM simultaneously.

All four studies described relevant interventions for classifying patients with DM who had complications and symptoms of CVI. The main methodologies that were effective for differentiating participants with this pathological condition were clinical analysis performed by specialists, combined with supplementary tests with diagnostic values.

For diagnostic criteria, clinical-etiologic-anatomopathological classification (CEAP) scores were combined with supplementary imaging exams. The analyses conducted by Forssgren and Nelzén^19^ lasted from 3 to 9 months, of which the main diagnostic methods were ultrasonography with color Doppler (USD) and portable Doppler, which confirmed 94 cases of CVI among 246 analyzed individuals who had leg ulcers. Matic et al. conducted duplex scans of the veins of the lower limbs and measured the ankle-brachial index over a 1-year period.^3^ This approach identified a total of 112 individuals with mild forms of CVI, among the 324 recruited to the study.^3^ The use of magnetic resonance over a period of 7 years and 4 months to identify venous changes is a strong point of the studies undertaken by Meinel et al.^21^ The incidence of CVI detected by this method was 38 cases among 180 individuals diagnosed with or suspected of having peripheral arterial disease (PAD).

In studies by Babaei et al.,^20^ application of the CEAP classification over 10 years in 1,176 participants aged from 30 to 75 years who were initially unaware of the presence of CVI revealed CVI in 429 individuals. Mean follow-up of these participants was 7.7 years and the main comorbidities associated with DM were systemic arterial hypertension and smoking.^22^

## DISCUSSION

**Figure 2:**
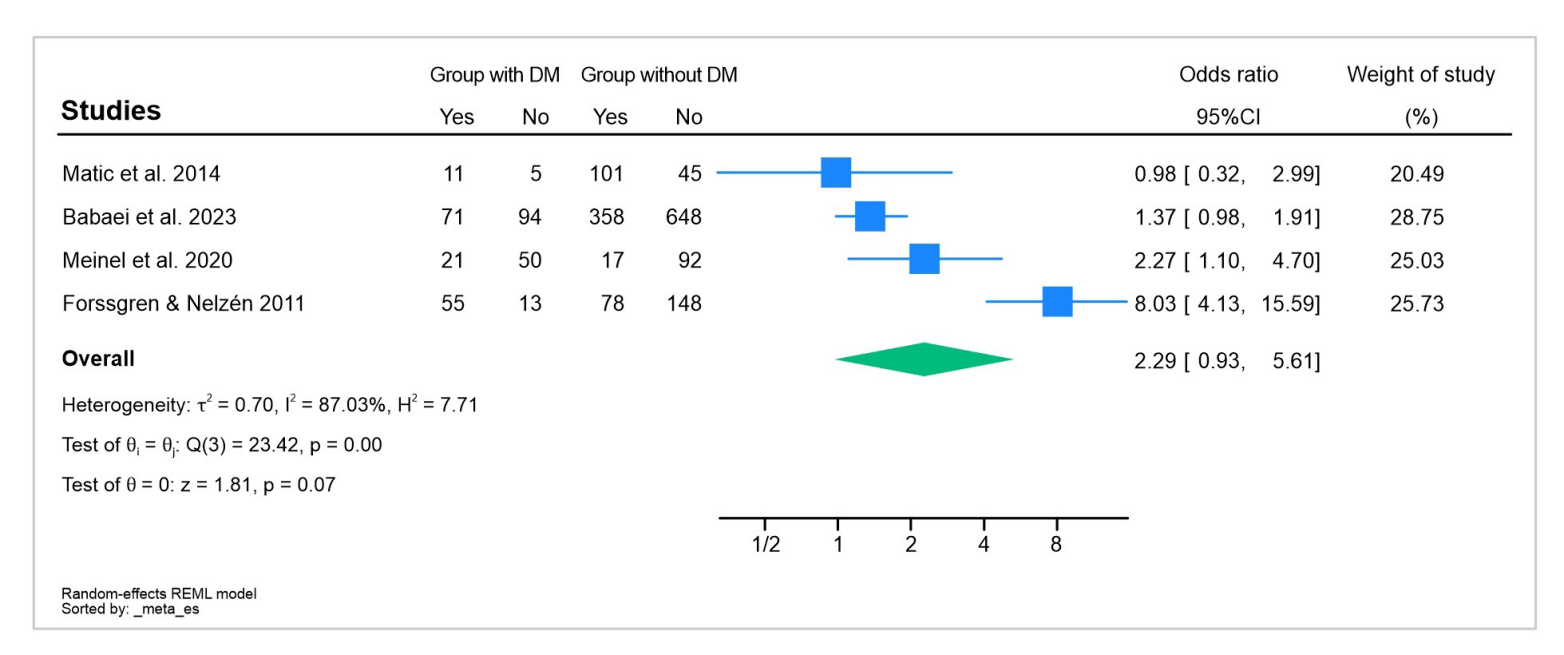
Odds ratios for occurrence of CVI in patients with DM. The forest plot does not demonstrate an association between CVI and DM. CVI = chronic venous insufficiency; DM = diabetes mellitus.

This systematic review is innovative in that it engages with a subject that has received little research attention to date - the association between CVI and DM. To the best of our knowledge, there are no similar studies in the databases consulted. The combined analysis of the results showed that there is no relationship between occurrence of CVI in people with DM and that the rate of occurrence of CVI among patients with diabetes is 56% (Figure 2).

**Figure 3:**
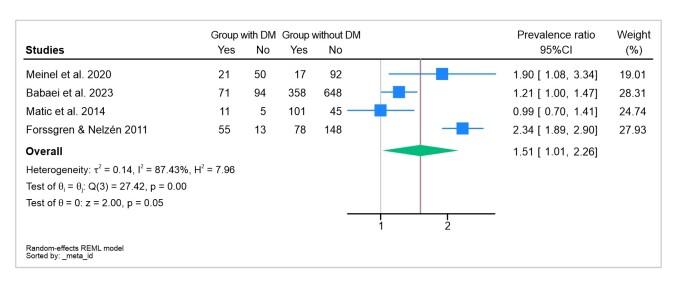
Prevalence ratios for occurrence of CVI in patients with DM. The forest plot shows that CVI is more associated with people with DM. CVI = chronic venous insufficiency; DM = diabetes mellitus.

The results vary depending on the analysis and effect measure adopted. It was observed that using the prevalence ratio as indicator resulted in a stronger association between CVI and people with DM, with a prevalence ratio value of 1.51 and a 95%CI of 1.01- 2.26. However, when the odds ratio was adopted as measure of effect, no significant association was observed, with an odds ratio value of 1.29 and a 95%CI of 0.93-5.61, as illustrated in Figure 3.

This systematic review included studies that described patients with DM who developed CVI. The majority were cross-sectional studies, based on medical charts from clinical follow-up of these patients. The diagnostic methods used included the CEAP classification,^20^ USD,^19^ duplex scan^3^ and magnetic resonance.^21^

Diagnosis and treatment of CVI are dependent on the CEAP classification (clinical signs, etiology, anatomic distribution, and pathophysiology), which forms a comprehensive, informative, and complete clinical picture of the functionality of the venous system. For a vascular surgeon to be able to diagnose CVI by physical examination, the patient must have evident clinical signs of varicosity of the venous vasculature, i.e. have a presentation with a CEAP clinical score greater than C2. The combination of tortuous veins with other symptoms such as edema, hyperpigmentation, ulcerations, and inflammatory signs is also sufficient for a diagnosis.^23^ Changes involving the deep vein system reduce the quality of this assessment, making supplementary imaging exams necessary for diagnostic confirmation.

USD is the additional imaging method most widely used to supplement incomplete investigations, because of its reproducibility, noninvasive nature, and the possibility of hemodynamic, anatomic, and physiological assessment of the deep or perforating vascular flow. Other imaging exams are also used to demonstrate presence of CVI, such as phlebography, photoplethysmography, magnetic resonance angiography and angiotomography. However, compared with ultrasonography, all of these methods are subject to difficulties with execution or low specificity. In patients with DM, it is even more important to assess CVI in great detail using the CEAP classification, because DM can exacerbate their venous complications. Studies indicate that DM can increase the risk and severity of CVI, due to the effects of hyperglycemia on blood vessels, causing inflammation and changes to the venous walls.^22,24^

Notwithstanding, certain factors are described in the literature as obstacles when venous reflux is assessed using USD in the deep and superficial systems. In particular, use of the accessory muscles in the buttocks while the sound waves are being administered, the position of the leg being tested, and weight bearing.^22^ Clinical assessment can be improved with maneuvers such as the Valsalva or mechanical compression of the calves, which improve diagnostic specificity, enabling assessment of venous reflux through incompetent valves.^25^

A variety of methods are used to identify the presence of venous incompetence. At the saphenofemoral junction, USD is used to obtain images of the venous system. Initially, the deep femoral vein is identified and its maximum venous flow is measured according to the residual volume detected by Doppler sonar. After stabilization of the spontaneous flow, the patient is instructed to cough deeply once. If reverse flow exceeding 500 ms is detected, the structure analyzed is diagnosed with venous insufficiency.^22^

**Figure 4:**
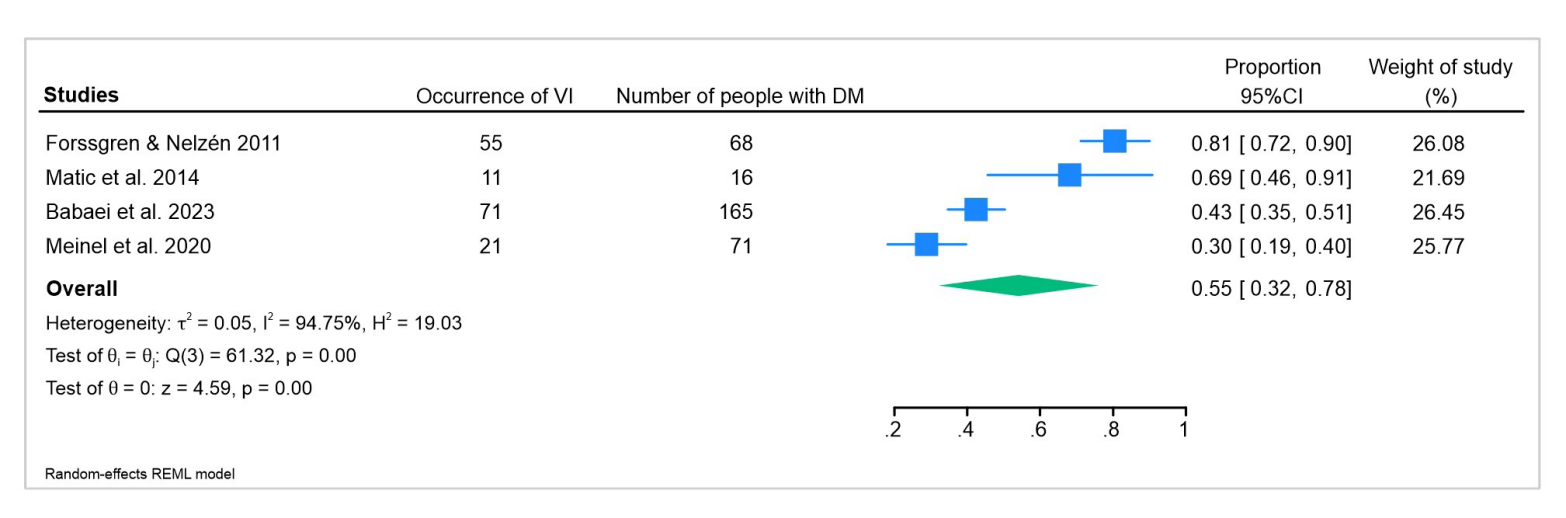
Occurrence of CVI in patients with DM, showing the relationship between the pathologies studied. Variations between the studies demonstrate possible increases in group sizes depending on the diagnostic approach. VI = venous insufficiency; CVI = chronic venous insufficiency; DM = diabetes mellitus.

The incidence rates of venous insufficiency among the diabetic patients in the studies reviewed were 81, 69, 43, and 30% (Figure 4). The study with the greatest occurrence included patients with a prior history of leg ulcers with clinical onset more than 6 months previously, i.e., the study analyzed patients to determine whether the primary cause of the ulcer was DM alone or the combination of DM with CVI. In turn, the 2014 study by Meinel et al. included patients with suspected PAD, in particular men aged 27 to 91 years. In the 2014 study by Matic et al., the patients recruited had been diagnosed with CVI or had a clinical suspicion of CVI, whereas there was no prior knowledge of CVI in the patients in the 2023 study by Babaei et al.

The studies analyzed risk factors associated with cardiovascular diseases, relating comorbidity not only with DM type 2 (DM2), but also with age, sex, smoking, inactivity, elevated body mass index, dyslipidemia, and systemic arterial hypertension, among other factors. They observed that intersections of these risk factors with DM were related to increased occurrence of CVI and its complications.

A study that assessed 3,072 individuals aged from 18 to 79 years, all with at least one leg symptom, such as tiredness, heaviness, pain, and others, found that 15.8% of them had CVI.^26^ In a different context, patients of both sexes aged 18 to 64 selected at random exhibited CVI prevalence rates of 9% in men and 7% in women.^27^ As such, when CVI prevalence is compared between patients with DM (56%) with the rate in studies that selected patients with symptoms^26^ or at random,^27^ it can be observed that prevalence is greater among patients with DM2.

Although the studies take a comprehensive approach to some aspects of the subject, their perspectives are limited. These difficulties emerge with respect to their investigation of the association between DM and CVI. The studies analyzed took different approaches. Only one of the studies employed a cohort design, while the others were all cross-sectional studies, which cannot be used to infer causality in the relationship between CVI and DM. They used diagnostic methods with considerable disparities in both sensitivity and specificity. The studies did not employ the internationally standardized criteria, which require use of USD combined with CEAP clinical findings.^6^ Diagnoses were made with a variety of methods, such as color USD, duplex scan, and magnetic resonance.

It is expected that observational studies will produce high values for heterogeneity in meta-analyses. Additionally, the small number of studies included precluded subgroup analyses.^28^

Results can vary depending on the analysis and measure of effect adopted. Using the prevalence ratio as indicator, we observed a stronger association between CVI and people with DM, with a prevalence ratio value of 1.51 and a 95%CI of 1.01-2.26. However, we had elected to use analysis of odds ratios as the measure of effect, and in this analysis we did not find a significant association.

The association between CVI and DM is a subject that has been little explored in the scientific literature, despite having important clinical implications. Although there is wide recognition of the vascular complications of diabetes, including micro and macroangiopathy, the direct relationship with CVI has not been clearly established. Studies suggest that the microvascular damage caused by DM could contribute to changes in venous function, potentially increasing the risk of developing CVI. However, as demonstrated in the present systematic review, the available evidence indicates that there is a high prevalence of CVI among diabetic patients, at around 56%, but does not confirm a clear causal association between the two conditions. This high prevalence could be related to factors in common, such as advanced age, obesity, and inactivity, which affect the development of both DM and CVI. Therefore, more studies are needed to elucidate whether DM is an independent risk factor for CVI or if the relationship between these conditions is mediated by other shared risk factors.

Given the inconsistent nature of the studies completed to date, it is essential that more prospective cohort studies be conducted to examine this relationship with greater rigor. Such studies should help to confirm or refute the hypothesis that CVI and DM2 can be considered connected comorbidities, yielding more solid evidence to guide clinical management of patients with these conditions.

## CONCLUSIONS

The incidence of CVI in patients with DM2 is an issue that is clinically relevant to understanding the development of chronic venous disease and its potential complications. It was observed that there was a 56% occurrence of venous vascular changes among patients with DM2 and that the prevalence ratio is 1.51 in favor of development of CVI in patients with DM. However, differences in the methodology, information, and approach of the available studies suggest that the association between CVI and DM2 cannot yet be firmly established on the basis of existing evidence. It is therefore necessary to conduct new prospective cohort studies with rigorous methodology and robust samples to elucidate this possible relationship.

## Supplementary Material

Supplementary material accompanies this paper.

**Supplementary Table 1:** Results for risk of bias of studies analyzed using the ROBINS-I tool.

This material is available as part of the online article from https://doi.org/10.1590/1677-5449.202500062

## References

[1] Muzy J, Campos MR, Emmerick I, Silva RS, Schramm JMA (2021). Prevalência de Diabetes mellitus e suas complicações e caracterização de lacunas na atenção à saúde a partir da triangulação de uma pesquisa. Cad Saude Publica.

[2] Branisteanu DE, Feodor T, Baila S, Mitea IA, Vittos O (2019). Impacto da doença venosa crônica na qualidade de vida: resultados do estudo de alarme venoso. Exp Ther Med.

[3] Matic M, Matic A, Djuran V, Gajinov Z, Prcic S, Golusin Z (2016). Frequência de doença arterial periférica em pacientes com insuficiência venosa crônica. Crescente Vermelho Iraniano Med J..

[4] Brasil (2013). Estratégias para o cuidado de pessoas com doenças crônicas: Diabetes Mellitus.

[5] Bertoluci MC, Forti AC, Pititto B A (2021). Diretriz da Sociedade Brasileira de Diabetes. Conectando Pessoas, 2021.

[6] Sociedade Brasileira de Angiologia e Cirurgia Vascular (2015). Insuficiência venosa crônica: diagnóstico e tratamento.

[7] Meissner MH, Khilnani NM, Labropoulos N (2021). The Symptoms-Varices-Pathophysiology classification of pelvic venous disorders: a report of the American Vein & Lymphatic Society International Working Group on Pelvic Venous Disorders. J Vasc Surg Distúrbio Linfático Venoso..

[8] Kikuchi R, Nhuch C, Drummond DAB (2023). Diretriz brasileira de doença venosa crônica da Sociedade Brasileira de Angiologia e Cirurgia Vascular. J Vasc Bras.

[9] Criqui MH, Jamosmos M, Fronek A (2003). Chronic venous disease in an ethnically diverse population: the San Diego population study. Am J Epidemiol.

[10] Carpentier PH, Maricq HR, Biro C, Ponçot-Makinen CO, Franco A (2004). Prevalência, fatores de risco e padrões clínicos de doenças venosas crônicas de membros inferiores: um estudo de base populacional na França. J Vasc Surg.

[11] Chiesa R, Marone EM, Limoni C, Volonté M, Schaefer E, Petrini O (2005). Insuficiência venosa crônica na Itália: o estudo de coorte de 24 cidades. Eur J Vasc Endovasc Surg.

[12] Jawien A, Grzela T, Ochwat A (2003). Prevalência de insuficiência venosa crônica em homens e mulheres na Polônia: estudo transversal multicêntrico em 40.095 pacientes. Flebologia..

[13] Rabe E, Pannier-Fischer F, Bromen K (2003). Bonner Venenstudie der Deutschen Gesellschaft für Phlebologie: Epidemiologische Untersuchung zur Frage der Häufigkeit und Ausprägung von chronischen Venenkrankheiten in der städtischen Und ländlichen Wohnbevölkerung. Flebologia..

[14] Wittens C, Davies AH, Bækgaard N (2015). Editor's choice - management of chronic venous disease: clinical practice guidelines of the European Society for Vascular Surgery (ESVS). Eur J Vasc Endovasc Surg.

[15] Brand FN, Dannenberg AL, Abbott RD, Kannel WB (1988). The epidemiology of varicose veins: the Framingham study. Am J Prev Med.

[16] Heit JÁ, Rooke TW, Silverstein MD (2001). Tendências na incidência de síndrome de estase venosa e úlcera venosa: um estudo de base populacional de 25 anos. J Vasc Surg.

[17] DATASUS (2024). Informações de saúde (TABNET).

[18] University of Bristol The ROBINS-E tool (Risk of bias in non-randomized studies - of exposures).

[19] Forssgren A, Nelzén O (2012). Alterações no espectro etiológico de úlceras de perna após uma intervenção em larga escala em uma população geográfica definida na Suécia. Eur J Vasc Endovasc Surg.

[20] Babaei M, Afrooghe A, Rafati A (2023). Prevalência e fatores associados à doença venosa crônica entre a população urbana iraniana moderna. J Vasc Surg Distúrbios Venosos e Linfáticos..

[21] Meinel FG, Ammermann F, Beller E (2020). Insuficiência venosa crônica concomitante em pacientes com doença arterial periférica: insights da angiografia por ressonância magnética. J Vasc Surg Distúrbios Venosos e Linfáticos..

[22] Singh TP, Velu RB, Quigley F, Golledge J (2022). Associação de doença venosa crônica com eventos cardiovasculares adversos maiores. J Vasc Surg Distúrbios Venosos e Linfáticos..

[23] Mani R, Yarde S, Edmonds M (2011). Prevalência de incompetência venosa profunda e anormalidades microvasculares em pacientes com diabetes mellitus. Jornal Internacional de Feridas nas Extremidades Inferiores.

[24] Cole W (2022). Chronic venous insufficiency and the diabetic patient: is there a connection?. Lower Extremity Review.

[25] Cires-Drouet R, Fangyang S, Rosenberger L (2020). Alta prevalência de doença venosa crônica entre profissionais de saúde nos Estados Unidos. J Vasc Surg Venous Lymphat Disord.

[26] Wrona M, Jöckel KH, Pannier F, Bock E, Hoffmann B, Rabe E (2015). Associação de distúrbios venosos com sintomas nas pernas: resultados do estudo da veia de Bonn 1. Revista Europeia de Cirurgia Vascular e Endovascular..

[27] Evans CJ, Fowkes FG, Ruckley CV, Lee AJ (1999). Prevalence of varicose veins and chronic venous insufficiency in men and women in the general population: Edinburgh Vein Study. J Epidemiol Community Health..

[28] Pereira MG, Galvão TF (2014). Etapas de busca e seleção de artigos em revisões sistemáticas da literatura. Epidemiol Serv Saude.

