# Supplementary material for: Systematic review and meta-analysis of the impact of diabetes mellitus on chronic venous insufficiency
Source: J Vasc Bras. 2025 Jul 28;24:e20250006. doi: 10.1590/1677-5449.202500062 (PMC12315575; doi:10.1590/1677-5449.202500062)
Supplement: Translation

# Translation: Revisão sistemática e metanálise sobre o impacto do diabetes melito na insuficiência venosa crônica

**DOI:** 10.1590/1677-5449.202500061

**Authors:** Alex Carlos Ferreira de Castro, Anderson Veiga Barbosa, David Fonseca Lima, João Vitor Rodrigues Vidal, José Maciel Caldas Reis, Saul Rassy Carneiro

**Affiliations:** 1 Universidade Federal do Pará UFPA, Belém, PA, Brasil.; 2 Universidade do Estado do Pará UEPA, Belém, PA, Brasil.

**Keywords:** complicações vasculares, diabetes melito tipo 2, ocorrência

## Abstract

Esta revisão sistemática com metanálise pesquisou a ocorrência de insuficiência venosa crônica (IVC) em pacientes com diabetes melito tipo 2 (DM2). A pergunta norteadora utilizou a estratégia PECOT (P: população; E: exposição; C: comparação; O: *outcome* [desfecho]; T: tipo de estudo), e o risco de viés foi analisado com a ferramenta ROBIS-I. A análise incluiu quatro estudos, e os resultados mostraram que a ocorrência de IVC em pacientes com DM2 foi de 55%, com uma razão de prevalência de 1,51 (IC95% 1,01-2,26). Assim, a prevalência de IVC é maior em pessoas com DM2 em comparação com a população sem a doença. No entanto, esta revisão destacou limitações, como a escassez de estudos longitudinais e a disparidade nos métodos de diagnóstico de IVC entre os estudos, evidenciando a necessidade de mais pesquisas sobre o tema.

## INTRODUÇÃO

O diabetes melito (DM) e a insuficiência venosa crônica (IVC) representam problemas de saúde pública de crescente relevância, dada a sua alta prevalência e as complexas interações fisiopatológicas que compartilham^1-3^. O DM é definido como uma disfunção crônica do metabolismo da glicose, resultante de anormalidades na ação ou produção de insulina, estando associado a diversas complicações sistêmicas, incluindo doenças cardiovasculares, nefropatias e neuropatias, que afetam adversamente a qualidade de vida dos indivíduos^4^. As complicações microvasculares e macrovasculares associadas ao DM não resultam apenas da hiperglicemia prolongada, mas também de mecanismos subjacentes complexos, como inflamação, disfunção endotelial e estresse oxidativo, que contribuem para o dano vascular e a progressão da doença^1,4,5^.

A IVC é caracterizada pela incapacidade funcional das veias em promover o retorno adequado do sangue dos membros inferiores para o coração, resultando em sintomas como edema, dor e, em casos mais avançados, úlceras venosas^6,7^. A etiologia da IVC é multifatorial, envolvendo fatores de risco como idade avançada, obesidade, história familiar e períodos prolongados de imobilidade nas posições ortostática ou sentada^7,8^. Essa condição afeta milhões de pessoas em todo o mundo, com prevalência crescente em decorrência do envelhecimento populacional e consistência em vários países^8-14^. Estima-se que a prevalência mundial de IVC esteja entre 10 e 30% da população adulta, com maior frequência em mulheres do que em homens, em uma proporção aproximada de 2:1^9-14^. Registros epidemiológicos europeus e estadunidenses indicam que a prevalência de IVC em adultos de 30 a 70 anos varia de 5 a 15%, sendo que cerca de 1% dos casos evoluem para complicações ulcerativas, acometendo mais de 7 milhões de pessoas e sendo responsável por 70 a 90% das úlceras de membros inferiores^15,16^.

No Brasil, há uma escassez de estudos sobre a prevalência e incidência de IVC. No entanto, dados dos sistemas de informação em saúde do Sistema Único de Saúde (SUS), entre 2021 e 2024, indicam 359.603.424 registros de alterações vasculares relacionadas (CID-10: I82.9, I80.3 e I74.4)^17^.

Embora o DM e a IVC sejam condições frequentemente encontradas na prática clínica, há uma lacuna significativa na literatura sobre sua associação. Poucos estudos exploram comparativamente essas duas doenças, e há uma escassez de dados que examinem suas semelhanças e efeitos combinados no agravamento das disfunções do sistema circulatório. O objetivo desta revisão sistemática com metanálise é explorar a associação entre DM e IVC, investigando a incidência de IVC em pacientes com DM e elucidando as possíveis interações patológicas que contribuem para o desenvolvimento e progressão dessas condições.

## MÉTODOS

Esta revisão sistemática com metanálise seguiu as recomendações do Preferred Reporting Items for Systematic Reviews and Meta-Analyses (PRISMA) e está registrada no banco de dados de revisões sistemáticas do International Prospective Register of Systematic Reviews (PROSPERO), sob o ID: CRD42024500961, a fim de evitar duplicação não intencional de publicação. Os critérios de elegibilidade consistiram em estudos observacionais transversais, caso-controle e coorte, sem restrição quanto ao período de publicação. A questão de pesquisa foi formulada usando os critérios PECOT (P: população; E: exposição; C: comparação; O: *outcome* [desfecho]; T: tipo de estudo), sendo: população, adultos maiores de 18 anos; exposição, pessoas com DM; comparação, pessoas sem DM; desfecho, progressão para insuficiência venosa crônica; tipo de estudo, estudos observacionais transversais, caso-controle ou coorte.

Foram excluídos estudos sem comparador, revisões de literatura e escopo e séries de casos. Não houve limitação quanto ao idioma de publicação. A identificação dos estudos foi realizada nas bases de dados PubMed, EMBASE e LILACS. As buscas foram combinadas com todos os descritores (''Diabetes Mellitus'', ''complicações'', ''Insuficiência venosa crônica'', ''comorbidades'' e ''associação''), utilizando os operadores booleanos "AND" e/ou "OR", e realizadas de forma independente por dois revisores, sendo posteriormente comparadas. Os artigos duplicados foram excluídos e os demais seguiram para a análise de títulos e resumos, onde aqueles que atendiam aos critérios de inclusão foram selecionados para a etapa de leitura completa da publicação. Qualquer divergência foi discutida para estabelecer um consenso, com a participação de um terceiro avaliador.

Os dados foram tabulados e analisados no *software* STATA 18.0 para modelos de efeitos aleatórios, e as medidas de efeito foram obtidas pela ocorrência dos efeitos na razão de chances. A proporção de ocorrência de IVC foi calculada com intervalo de confiança de 95%. A heterogeneidade estatística dos efeitos do tratamento entre os estudos foi avaliada pelo índice de inconsistência (I^2^), estatística TAU2 e índice Q.

O risco de viés foi avaliado utilizando a ferramenta ROBINS-I, recomendada pela colaboração Cochrane (Tabela Suplementar 1, somente on-line). Foram considerados os seguintes domínios: viés por fatores de confusão, viés na seleção dos participantes do estudo, viés na classificação das intervenções, viés devido a desvios das intervenções pretendidas, viés devido à falta de dados, viés na mensuração dos desfechos, viés na seleção dos desfechos relatados e viés geral. A qualidade das evidências foi analisada de acordo com o sistema Grading of Recommendations Assessment, Development and Evaluation (GRADE), sendo realizada de forma independente por dois avaliadores. As discordâncias foram discutidas até que se chegasse a um consenso, através da participação de um terceiro avaliador^18^. Os riscos de viés dos estudos analisados estão disponíveis na Tabela Suplementar 1.

## RESULTADO

A estratégia de busca de dados on-line revelou 1.183 artigos, com aplicabilidade das palavras-chave ''Diabetes Mellitus'', ''complicações'', ''Insuficiência venosa crônica'', ''comorbidades'' e ''associação''. Desses, após extensas implementações iniciais de triagem por meio da leitura de títulos e resumos, 1.165 artigos foram excluídos. Para leitura completa, foram selecionados 18 artigos. Deste montante, 10 artigos foram excluídos por não apresentarem destaque central às doenças estudadas. Dos oito estudos restantes, dois foram excluídos por incluírem apenas pacientes com IVC, não tendo sido realizada triagem para diferenciação entre os grupos. Além disso, um artigo foi excluído por apresentar dados estatísticos escassos entre o número de pacientes com DM e a proporção de acometidos por insuficiência venosa, sendo, portanto, de pouca relevância para o estudo. Por fim, um artigo foi retirado deste trabalho devido a dificuldades de acesso ao texto completo, após uma solicitação ao autor principal não ter sido bem-sucedida.

No total, quatro estudos foram incluídos nesta pesquisa, sendo três transversais e apenas um de coorte prospectiva. No entanto, todos compararam efetivamente a presença de IVC em pacientes com DM. O diagrama PRISMA é apresentado na Figura 1.

**Figura 1:** Fluxograma PRISMA (Preferred Reporting Items for Systematic Reviews and Meta-Analyses) dos estudos incluídos, mostrando a identificação dos estudos, o estágio em que foram excluídos e os motivos da exclusão. IVC = insuficiência venosa crônica; *considere, se possível, relatar o número de registros identificados em cada base de dados ou registro pesquisado (em vez do número total em todos os bancos de dados/registros); **se foram utilizadas ferramentas de automação, indique quantos registros foram excluídos por um humano e quantos foram excluídos porferramentas de automação.

O período de análise abordado por esses estudos foi entre os anos de 2009 e 2023, com centros de estudo na Sérvia, Suécia, Irã e Alemanha. O estudo realizado por Matic et al.^3^ incluiu 174 pacientes submetidos à análise. Destes, nove foram excluídos por falta de consentimento ou incompletude dos prontuários, restando um total de 162 pacientes analisados, dos quais 11 apresentavam IVC e DM. Outro estudo, realizado por Forssgren e Nelzén^19^, apresentou resultados semelhantes ao anterior, com uma amostra inicial de 621 participantes. Após a exclusão dos testes e retirada do acompanhamento, 207 participantes foram analisados. Destes, 68 tinham DM e oito tinham DM e IVC concomitantes. As análises de Babaei et al.^20^ demonstraram dados significativos, incluindo um total de 1.176 participantes. As evidências estatísticas mostraram que 165 tinham DM, dos quais 71 também tinham IVC. Ao mesmo tempo, os dados apresentados por Meinel et al.^21^ mostraram perspectivas relevantes, com 71 pacientes com DM, dos quais 21foram acometidos simultaneamente por IVC e DM.

Todos os estudos apresentaram intervenções relevantes para qualificar pacientes com DM que apresentavam complicações e sintomas de IVC. As principais metodologias que se mostraram eficazes na diferenciação dos participantes acometidos por essa condição patológica foram a análise clínica realizada por especialistas, associada a exames complementares com valores diagnósticos.

Para os critérios diagnósticos, os valores da classificação clínico-etiológico-anatomopatológica (CEAP) foram associados a exames complementares de imagem. As abordagens de Forssgren e Nelzén^19^ duraram de 3 a 9 meses, sendo os principais métodos diagnósticos a ultrassonografia com Doppler (USD) colorido e o Doppler portátil, que indicaram 94 confirmações de IVC entre os 246 indivíduos analisados que apresentavam úlceras de perna. O *duplex scan* das veias dos membros inferiores e o índice tornozelo-braquial foram medidos durante 1 ano nos dados fornecidos por Matic et al.^3^. Essas abordagens identificaram um total de 112 indivíduos com formas leves de IVC, entre os 324 adicionados ao estudo^3^. Destaca-se ainda o uso da ressonância magnética nos estudos realizados por Meinel et al.^21^, ao longo de um período de 7 anos e 4 meses, como forma de verificar alterações venosas. A ocorrência de IVC indicada por esse método foi de 38 casos entre os 180 indivíduos diagnosticados ou suspeitos de doença arterial periférica (DAP).

Nos estudos de Babaei et al.^20^, a aplicação da classificação CEAP ao longo de 10 anos em 1.176 participantes, que inicialmente desconheciam a presença de IVC e tinham entre 30 e 75 anos, demonstrou a presença de IVC em 429 indivíduos. O seguimento dos participantes teve média de 77 anos, sendo as principais comorbidades associadas o DM, a hipertensão arterial sistêmica e o tabagismo^22^.

## DISCUSSÃO

A presente revisão sistemática é inovadora por abordar um tema ainda pouco pesquisado, que é a associação entre IVC e DM. Até onde se tem conhecimento, não há estudo semelhante nas bases de dados consultadas. A análise conjunta dos resultados mostrou que não há relação entre a ocorrência de IVC em pessoas com DM, e que a ocorrência de IVC em diabéticos é de 56% (Figura 2).

**Figura 2:** Razão de chances de ocorrência de IVC em pacientes com DM. O gráfico em floresta não demonstra associação entre IVC e DM. IVC = insuficiência venosa crônica; DM = diabetes melito.

Os resultados variaram conforme a análise e medida de efeito adotadas. Quando se utilizou a razão de prevalência como indicador, observou-se que houve maior associação entre IVC e pessoas com DM, com valor de 1,51 e IC95% (1,01- 2,26). No entanto, quando se optou por adotar a razão de chances como medida de efeito, não foi encontrada associação significativa, com valor de 1,29 e IC95% (0,93-5,61), conforme demonstrado na Figura 3.

**Figura 3:** Razão de prevalência de ocorrência de IVC em pacientes com DM. O gráfico em floresta mostra que o IVC está mais associado às pessoas com DM. IVC = insuficiência venosa crônica; DM = diabetes melito.

Na presente revisão sistemática, foram considerados estudos que relacionaram pacientes com DM que desenvolveram IVC. A maioria desses estudos foi do tipo transversal, baseada em prontuários médicos do acompanhamento clínico desses pacientes. Os métodos diagnósticos utilizados incluíram classificação CEAP^20^, USD^19^, *duplex scan* ^3^ e ressonância magnética^21^.

O diagnóstico e o tratamento da IVC dependem da classificação CEAP (sinais clínicos, etiologia, distribuição anatômica, fisiopatologia), que fornece um quadro clínico abrangente, didático e completo da funcionalidade do sistema venoso. Para que os cirurgiões vasculares possam diagnosticar a IVC por meio do exame físico, é necessário evidenciar sinais clínicos de varicosidade da vasculatura venosa, ou seja, apresentar um quadro com escore acima de C2 da classificação CEAP. A associação de veias tortuosas com outros sintomas como edema, hiperpigmentação, ulcerações e sinais flogísticos de inflamação também são suficientes para o diagnóstico^23^. Alterações no sistema venoso profundo reduzem a qualidade dessa avaliação, exigindo o uso de exames complementares de imagem para elucidação diagnóstica.

A USD é o método adicional mais comumente aplicado em casos de investigações incompletas, devido à sua reprodutibilidade, caráter não invasivo e à possibilidade de avaliação hemodinâmica, anatômica e fisiológica do fluxo vascular profundo ou perfurante. Outros exames de imagem também são utilizados para evidenciar IVC, como flebografia, fotopletismografia, angiografia por ressonância magnética e angiotomografia. Porém, todos esses métodos apresentam dificuldade de execução ou baixa especificidade em comparação com a ultrassonografia. No contexto do DM, é ainda mais importante avaliar minuciosamente os pacientes com IVC utilizando a classificação CEAP, pois o DM pode piorar as complicações venosas. Estudos indicam que o DM pode aumentar o risco e a gravidade da IVC, devido aos efeitos da hiperglicemia nos vasos sanguíneos, os quais causam inflamação e alterações nas paredes venosas^22,24^.

No entanto, quando o refluxo venoso é avaliado por USD nos sistemas profundo e superficial, alguns fatores de interferência são relatados na literatura como potenciais obstáculos. Destacam-se o uso da musculatura acessória das nádegas durante a aplicação do sonar, o posicionamento das pernas a serem testadas e a sustentação de peso^22^. Para melhorar a avaliação clínica, manobras como Valsalva ou compressão mecânica das panturrilhas permitem maior especificidade diagnóstica, possibilitando a avaliação do refluxo venoso em valvas incompetentes^25^.

Os métodos utilizados para identificar a presença de incompetência venosa são variados. A USD na junção safeno-femoral é utilizada para obter imagens do sistema venoso. Inicialmente, localiza-se a veia femoral profunda e, em seguida, seu fluxo venoso máximo é medido através do volume residual detectado pelo sonar Doppler. Após a estabilização do fluxo espontâneo, o paciente é solicitado a realizar uma única tosse profunda. Se houver reversão do fluxo superior a 500 ms, o diagnóstico de insuficiência venosa é feito na estrutura analisada^22^.

A incidência de insuficiência venosa em pacientes diabéticos nos estudos foi de 81, 69, 43 e 30% (Figura 4). O estudo com a maior ocorrência incluiu pacientes com história prévia de úlcera de perna, com evolução clínica superior a 6 meses, ou seja, o estudo analisou os pacientes para distinguir se a causa primária da úlcera era o DM isoladamente ou a associação de DM com IVC. Já no estudo de Meinel et al, de 2014, foram incluídos pacientes com suspeita de DAP, especialmente homens com idades entre 27 e 91 anos. No estudo de Matic et al., de 2014, os pacientes incluídos foram diagnosticados ou tinham suspeita clínica de IVC, enquanto no estudo de Babaei et al., de 2023, não havia conhecimento prévio sobre pacientes com IVC.

**Figura 4:** Ocorrência de IVC em pacientes com DM mostrando relação entre as patologias abordadas. Variações entre os estudos demonstram possível amplificação dos grupos de acordo com a abordagem diagnóstica. IV = insuficiência venosa; IVC = insuficiência venosa crônica; DM = diabetes melito.

Os estudos avaliaram os fatores de risco associados às doenças cardiovasculares, relacionando a comorbidade não apenas ao DM tipo 2 (DM2), mas também à idade, sexo, tabagismo, sedentarismo, índice de massa corporal elevado, dislipidemia, hipertensão arterial sistêmica, entre outros. Assim, observou-se que as intersecções dos fatores de risco com o DM foram relacionadas a uma maior ocorrência de desenvolvimento de IVC e suas complicações.

Em estudo que avaliou 3.072 indivíduos com idades entre 18 e 79 anos, todos com pelo menos um sintoma nas pernas, como cansaço, peso, dor, entre outros, verificou-se que 15,8% dos participantes apresentavam IVC^26^. Em outro cenário, pacientes selecionados aleatoriamente, com idades entre 18 e 64 anos, de ambos os sexos, apresentaram prevalência de IVC de 9% em homens e 7% em mulheres^27^. Nesse sentido, quando comparada a prevalência de IVC entre pacientes com DM (56%) com outros estudos que selecionaram pacientes por sintomas^26^ ou aleatoriamente^27^, é possível observar uma maior prevalência em pacientes com DM2.

Embora os estudos abordem amplamente aspectos específicos do tema, apresentam limitações em suas perspectivas. Ao investigar a associação entre DM e IVC, os produtos apresentam dificuldades. Os estudos analisados apresentaram diferentes perfis em suas formas de abordagem. No total, apenas um dos estudos utilizou um modelo de coorte, enquanto os demais foram estudos transversais, não sendo possível inferir causalidade para a associação entre IVC e DM. Foram utilizados métodos diagnósticos com grande disparidade, tanto na sensibilidade quanto na especificidade. Os estudos não seguiram os critérios padronizados internacionalmente, que preconizam o uso da USD associado aos achados clínicos do CEAP^6^. Para o diagnóstico, foram utilizadas diferentes abordagens, como a USD colorido, o *duplex scan* e a ressonância magnética.

Em estudos observacionais, espera-se que altos valores de heterogeneidade sejam encontrados durante a metanálise. Ademais, o pequeno número de estudos incluídos dificultou a análise dos subgrupos^28^.

Os resultados podem variar dependendo da análise e medida de efeito adotadas. Se a razão de prevalência for utilizada como indicador, verificaremos que há maior associação entre IVC e pessoas com DM, com valor de 1,51 e IC95% (1,01-2,26). No entanto, optou-se por apresentar a análise da razão de chances como medida de efeito, e neste caso, não identificamos associação significativa.

A associação entre IVC e DM é um tema que, embora ainda pouco explorado na literatura científica, tem importantes implicações clínicas. Embora o diabetes seja amplamente reconhecido por suas complicações vasculares, incluindo micro e macroangiopatia, a relação direta com a IVC não está claramente estabelecida. Estudos sugerem que o dano microvascular causado pelo DM pode contribuir para alterações na função venosa, aumentando potencialmente o risco de desenvolver IVC. No entanto, as evidências disponíveis, conforme demonstrado na presente revisão sistemática, indicam que a prevalência de IVC em pacientes diabéticos é alta, ficando em torno de 56%, mas não confirmam uma associação causal clara entre as duas condições. Essa alta prevalência pode estar relacionada a fatores comuns, como idade avançada, obesidade e sedentarismo, que afetam tanto o desenvolvimento de DM quanto IVC. Portanto, mais pesquisas são necessárias para elucidar se o DM é um fator de risco independente para IVC ou se a relação entre essas condições é mediada por outros fatores de risco compartilhados.

Dada a natureza inconsistente dos estudos até o momento, é essencial que mais estudos de coorte prospectivos sejam conduzidos para examinar essa relação com mais rigor. Esses estudos podem ajudar a confirmar ou refutar a hipótese de que a IVC e o DM2 podem ser considerados comorbidades articulares, fornecendo evidências mais sólidas para orientar o manejo clínico de pacientes com essas condições.

## CONCLUSÃO

A incidência de IVC em pacientes com DM2 representa um aspecto clinicamente relevante para a compreensão do desenvolvimento da doença venosa crônica e suas potenciais complicações. Verificou-se que há uma ocorrência de 56% de alterações vasculares venosas em pacientes com DM2, e que a razão de prevalência é de 1,51 a favor do desenvolvimento de IVC em pacientes com DM. No entanto, as disparidades metodológicas, de informação e de abordagem entre os estudos existentes sugerem que a associação entre IVC e DM2 ainda não pode ser firmemente estabelecida com base nas evidências atuais. Portanto, é necessário que novos estudos de coorte prospectivos, com metodologia rigorosa e amostras robustas, sejam realizados para esclarecer essa possível relação.

## Material Suplementar

Este artigo acompanha material suplementar.

**Tabela Suplementar 1**: Resultado do risco de viés dos estudos analisados com a ferramenta ROBINS-I.

Este material está disponível como parte da versão online do artigo na página https://doi.org/10.1590/1677-5449.202500061
